# Otitis Media and Its Intracranial Complications: A Delicate Scenario

**DOI:** 10.7759/cureus.76561

**Published:** 2024-12-29

**Authors:** Mariana Lobo, Rafaela Lopes Freitas, Miguel Viana, Frederico Duarte, Sara Camões

**Affiliations:** 1 Internal Medicine, Pedro Hispano Hospital, Matosinhos Local Health Unit, Matosinhos, PRT; 2 Otolaryngology, Pedro Hispano Hospital, Matosinhos Local Health Unit, Matosinhos, PRT; 3 Infectious Disease, Pedro Hispano Hospital, Matosinhos Local Health Unit, Matosinhos, PRT

**Keywords:** cerebral abscess, cholesteatoma, intracranial complications, otitis media, subdural empyema

## Abstract

Intracranial complications of otitis media are rare but pose a significant risk of morbidity and mortality. We report a case of a 27-year-old man with cognitive impairment who presented with fever, right-sided otalgia, otorrhea, and vomiting for three days. His neurological examination was unremarkable, and a brain computed tomography (CT) revealed right-sided otomastoiditis without intraparenchymal lesions. Despite intravenous antibiotic therapy, the patient's condition deteriorated within 24 hours, with worsening fever, fluctuating consciousness, and signs of meningeal irritation. A follow-up computed tomography revealed tympanic erosion, right temporal cerebritis, and edema. The patient underwent tympanomastoidectomy; however, seven days later, a brain magnetic resonance imaging (MRI) showed a brain abscess and subdural empyema. Surgical drainage was performed, and the patient completed a 13-week course of antimicrobial therapy, achieving a favorable clinical and imaging outcome. This case highlights the importance of early diagnosis and intervention in improving the prognosis of patients with intracranial complications of otitis media.

## Introduction

Otitis media is an infection of the middle ear cavity that can occur at any age and present in acute or chronic forms [1]. Advances in antibiotics and vaccination have reduced its prevalence and associated complications [1-3]. However, the anatomical proximity of the ear to critical structures increases the risk of both extracranial and intracranial complications [1,4]. Intracranial complications include meningitis, cerebral and subdural abscesses, and venous sinus thrombosis, all of which carry significant morbidity and mortality [1,3,5].

In developed countries, intracranial complications are uncommon but still occur, with abscesses being particularly concerning (11.1% mortality) [1,2,5-7]. Management typically involves a combination of surgical intervention with prolonged intravenous antibiotic therapy [5]. Conservative treatment may be used for small abscesses or in patients who are not candidates for surgery [1]. Early diagnosis and treatment are crucial for favorable outcomes.

In this report, we present a case of a young man with a temporal abscess and subdural empyema. The first tomography scan did not show the complication, but in less than 24 hours, the patient presented with a worsening general condition. After surgery and prolonged antibiotic treatment, the patient presented with almost complete recovery.

## Case presentation

A 27-year-old man with cognitive impairment secondary to fetal alcohol syndrome and a history of radical left mastoidectomy for chronic otitis media with cholesteatoma presented to the emergency department with a three-day history of fever, right-sided otalgia, otorrhea, and vomiting. On examination, he was alert and hemodynamically stable with no neurological deficits, but purulent drainage was observed from the right ear. Blood tests revealed elevated inflammatory markers, and a non-contrast brain computed tomography (CT) scan showed right otomastoiditis without intraparenchymal lesions. Intravenous amoxicillin and clavulanate were initiated.

**Figure 1:**
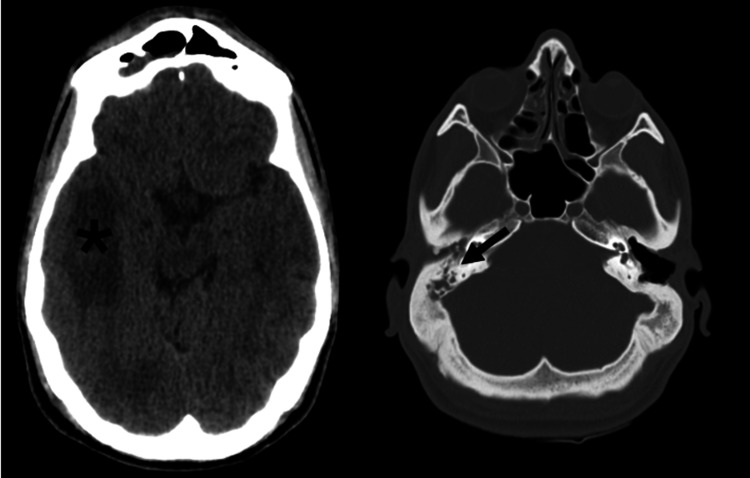
Brain CT with bone erosion (black arrow) and temporal cerebritis (*) with a possible abscess and edema. CT: computed tomography

Within 24 hours, the patient developed fluctuating consciousness, persistent fever (up to 39.8°C), and meningeal signs, including a positive Brudzinski sign. Lumbar puncture attempts were unsuccessful. A repeat CT scan revealed the erosion of the tympanum, attic wall, and stapes, along with right temporal cerebritis, edema, and a probable abscess (Figure 1). Tympanomastoidectomy was performed, revealing a cholesteatoma with cranial fossa communication. Pus samples yielded *Parvimonas micra* and *Eggerthella lenta*, although technical issues prevented antibiotic sensitivity testing. Antibiotic therapy was switched to cefepime and metronidazole, combined with intravenous dexamethasone to manage cerebral edema.

**Figure 2:**
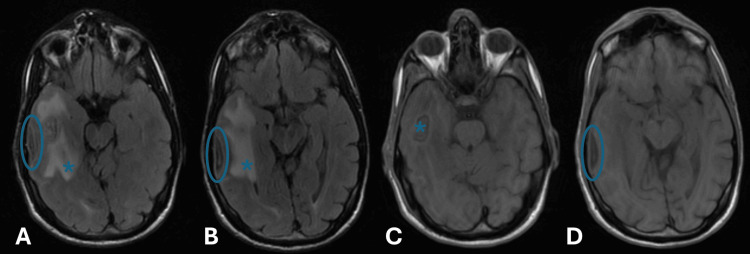
Brain MRI after one week of treatment showing an increase in abscess (*) and subdural empyema (blue circle). A and B are axial T2 flair slices; C and D are axial T1 slices. MRI: magnetic resonance imaging

A follow-up brain magnetic resonance imaging (MRI) 24 hours post-surgery showed a right temporal abscess and subdural empyema, prompting neurosurgical consultation. Initially, conservative management was chosen, but subsequent MRI revealed an enlarging abscess (26.8 × 19 mm) (Figure 2). An emergency craniotomy was performed to drain both the abscess and empyema. *Staphylococcus warneri*, a Gram-positive organism, was isolated with broad antibiotic sensitivity, allowing a switch back to cefepime and metronidazole.

**Figure 3:**
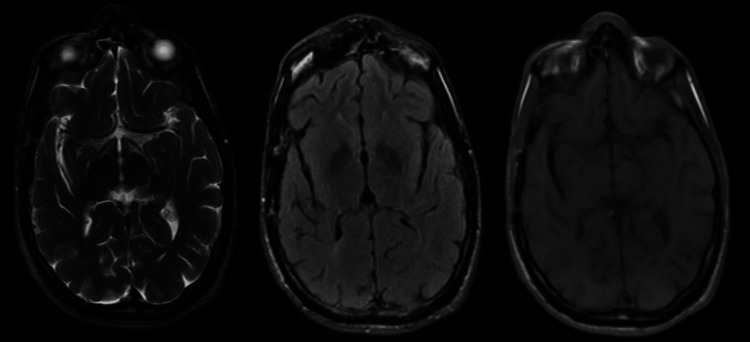
Brain MRI after 13 weeks of intravenous treatment and surgical drainage of the abscess. MRI: magnetic resonance imaging

The patient completed 13 weeks of intravenous antibiotics, with marked clinical and imaging improvement (Figure 3). MRI at the end of treatment revealed residual changes suggestive of metronidazole-induced encephalopathy. The patient returned to baseline cognitive function but experienced bilateral profound hearing loss. Follow-up over the subsequent year showed no signs of recurrence or complications.

## Discussion

Intracranial complications of otitis media are rare, with incidence estimates ranging from 0.04% to 3.5% [4]. Advances in antibiotics and vaccination have reduced their frequency, but emerging antimicrobial resistance and pathogen virulence may complicate cases [5-7]. Factors such as cholesteatoma, present in this case, significantly increase the risk of complications due to bone destruction and direct infection spread [2,3,8,9]. It is important to note that patients often present with multiple complications [9].

Brain abscesses, among the most common intracranial complications, may even account for around 50% of cases of intracranial complications [1,2,9], often resulting from direct infection spread or retrograde thrombophlebitis [4]. They frequently localize to the temporal lobe due to its proximity to the ear [1,2,5,6,9,10]. Without prompt treatment, these lesions can rupture, causing fulminant meningitis or brain herniation [4].

This case highlights diagnostic challenges, as the initial non-contrast CT scan failed to detect early abscess formation. A high index of suspicion and early imaging, preferably with contrast, are essential when otitis media presents with systemic or neurological symptoms.

Treatment typically involves early mastoidectomy to control the infectious focus, combined with prolonged intravenous antibiotics [1-6,9,10]. The surgical drainage of abscesses is performed based on size and progression [2,9]. In this case, mastoidectomy was performed first, with abscess drainage delayed until its progression was confirmed.

Unusual pathogens such as *Parvimonas micra* and *Eggerthella lenta* were isolated, reflecting the complexity of the microbiological profile in such cases. Prolonged antibiotic therapy is critical, with treatment durations often exceeding 6-8 weeks [4].

Despite advances, intracranial complications carry significant risks, with 30% of patients experiencing long-term sequelae such as hearing loss, as seen in this case [4,7].

## Conclusions

While advances in medical management have reduced the frequency of complications associated with otitis media, delayed diagnoses remain a challenge. This case underscores the importance of clinical vigilance, multidisciplinary care, and timely intervention to optimize outcomes. Ongoing awareness and early imaging are essential for managing these complex cases, preventing long-term sequelae, and improving survival rates.
